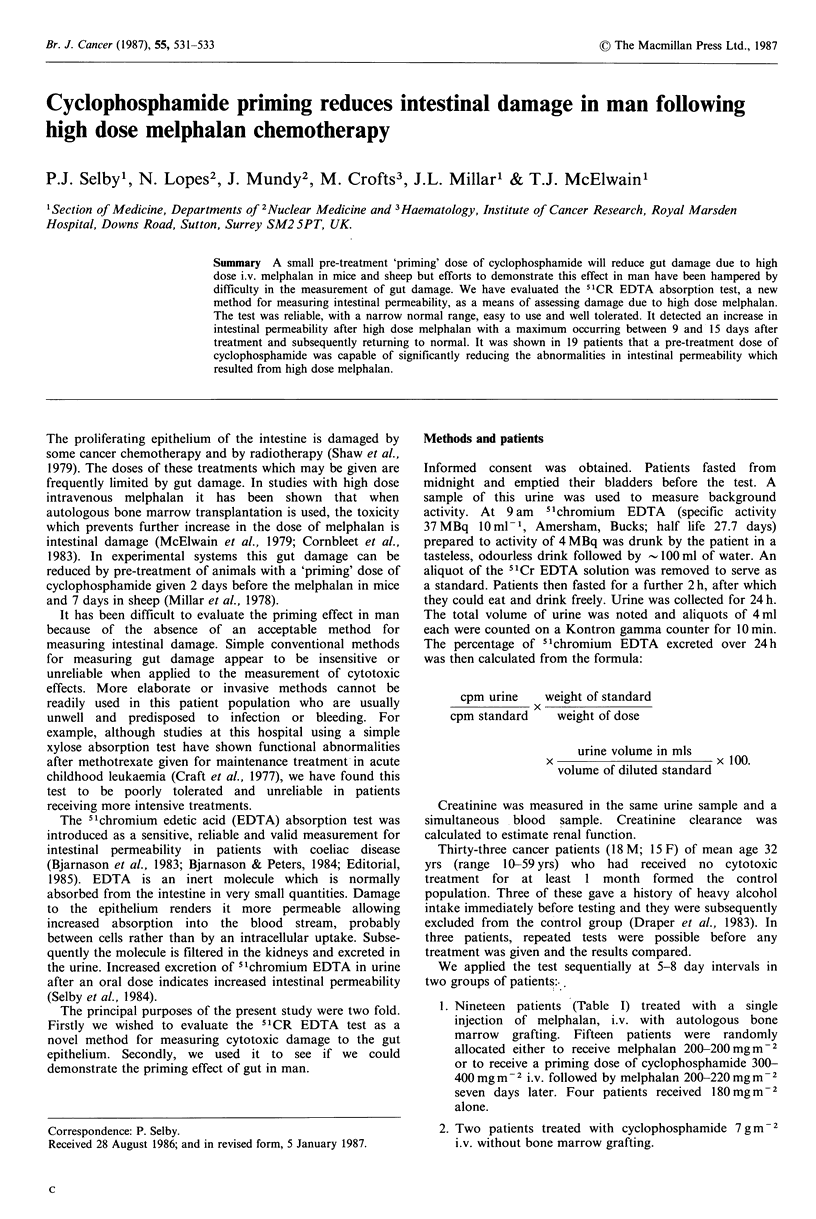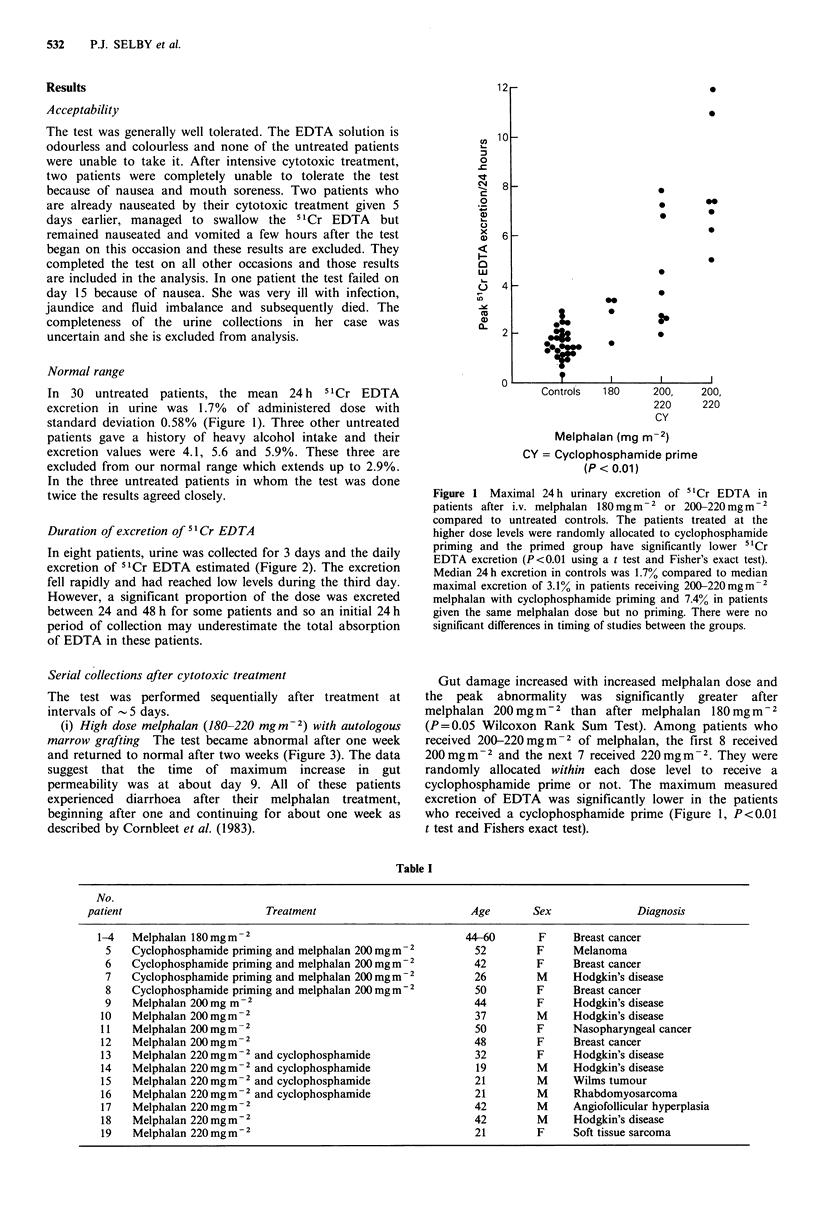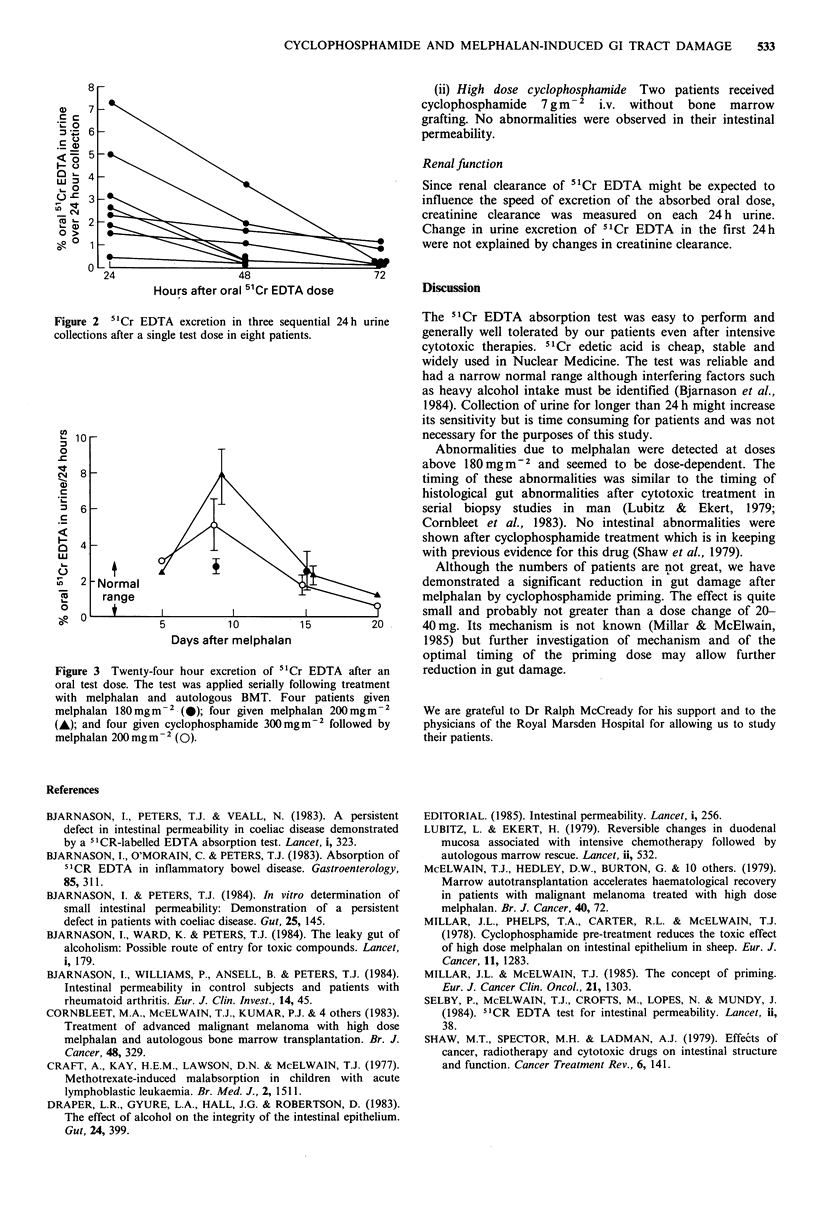# Cyclophosphamide priming reduces intestinal damage in man following high dose melphalan chemotherapy.

**DOI:** 10.1038/bjc.1987.108

**Published:** 1987-05

**Authors:** P. J. Selby, N. Lopes, J. Mundy, M. Crofts, J. L. Millar, T. J. McElwain

## Abstract

A small pre-treatment 'priming' dose of cyclophosphamide will reduce gut damage due to high dose i.v. melphalan in mice and sheep but efforts to demonstrate this effect in man have been hampered by difficulty in the measurement of gut damage. We have evaluated the 51CR EDTA absorption test, a new method for measuring intestinal permeability, as a means of assessing damage due to high dose melphalan. The test was reliable, with a narrow normal range, easy to use and well tolerated. It detected an increase in intestinal permeability after high dose melphalan with a maximum occurring between 9 and 15 days after treatment and subsequently returning to normal. It was shown in 19 patients that a pre-treatment dose of cyclophosphamide was capable of significantly reducing the abnormalities in intestinal permeability which resulted from high dose melphalan.


Br. J. Cancer (1987), 55, 531–533 © The Macmillan Press Ltd., 1987

### Cyclophosphamide priming reduces intestinal damage in man following high dose melphalan chemotherapy

P.J. Selby[1], N. Lopes[2], J. Mundy[2], M. Crofts[3], J.L. Millar[1] & T.J. McElwain[1]

[1]*Section of Medicine, Departments of* [2]*Nuclear Medicine and* [3]*Haematology, Institute of Cancer Research, Royal Marsden Hospital, Downs Road, Sutton, Surrey SM2 5PT, UK.*

**Summary** A small pre-treatment 'priming' dose of cyclophosphamide will reduce gut damage due to high dose i.v. melphalan in mice and sheep but efforts to demonstrate this effect in man have been hampered by difficulty in the measurement of gut damage. We have evaluated the $^{51}$CR EDTA absorption test, a new method for measuring intestinal permeability, as a means of assessing damage due to high dose melphalan. The test was reliable, with a narrow normal range, easy to use and well tolerated. It detected an increase in intestinal permeability after high dose melphalan with a maximum occurring between 9 and 15 days after treatment and subsequently returning to normal. It was shown in 19 patients that a pre-treatment dose of cyclophosphamide was capable of significantly reducing the abnormalities in intestinal permeability which resulted from high dose melphalan.

The proliferating epithelium of the intestine is damaged by some cancer chemotherapy and by radiotherapy (Shaw *et al.*, 1979). The doses of these treatments which may be given are frequently limited by gut damage. In studies with high dose intravenous melphalan it has been shown that when autologous bone marrow transplantation is used, the toxicity which prevents further increase in the dose of melphalan is intestinal damage (McElwain *et al.*, 1979; Cornbleet *et al.*, 1983). In experimental systems this gut damage can be reduced by pre-treatment of animals with a 'priming' dose of cyclophosphamide given 2 days before the melphalan in mice and 7 days in sheep (Millar *et al.*, 1978).

It has been difficult to evaluate the priming effect in man because of the absence of an acceptable method for measuring intestinal damage. Simple conventional methods for measuring gut damage appear to be insensitive or unreliable when applied to the measurement of cytotoxic effects. More elaborate or invasive methods cannot be readily used in this patient population who are usually unwell and predisposed to infection or bleeding. For example, although studies at this hospital using a simple xylose absorption test have shown functional abnormalities after methotrexate given for maintenance treatment in acute childhood leukaemia (Craft *et al.*, 1977), we have found this test to be poorly tolerated and unreliable in patients receiving more intensive treatments.

The $^{51}$chromium edetic acid (EDTA) absorption test was introduced as a sensitive, reliable and valid measurement for intestinal permeability in patients with coeliac disease (Bjarnason *et al.*, 1983; Bjarnason & Peters, 1984; Editorial, 1985). EDTA is an inert molecule which is normally absorbed from the intestine in very small quantities. Damage to the epithelium renders it more permeable allowing increased absorption into the blood stream, probably between cells rather than by an intracellular uptake. Subsequently the molecule is filtered in the kidneys and excreted in the urine. Increased excretion of $^{51}$chromium EDTA in urine after an oral dose indicates increased intestinal permeability (Selby *et al.*, 1984).

The principal purposes of the present study were two fold. Firstly we wished to evaluate the $^{51}$CR EDTA test as a novel method for measuring cytotoxic damage to the gut epithelium. Secondly, we used it to see if we could demonstrate the priming effect of gut in man.

Correspondence: P. Selby.
Received 28 August 1986; and in revised form, 5 January 1987.

### Methods and patients

Informed consent was obtained. Patients fasted from midnight and emptied their bladders before the test. A sample of this urine was used to measure background activity. At 9 am $^{51}$chromium EDTA (specific activity 37 MBq 10 ml$^{-1}$, Amersham, Bucks; half life 27.7 days) prepared to activity of 4 MBq was drunk by the patient in a tasteless, odourless drink followed by $\sim$100 ml of water. An aliquot of the $^{51}$Cr EDTA solution was removed to serve as a standard. Patients then fasted for a further 2 h, after which they could eat and drink freely. Urine was collected for 24 h. The total volume of urine was noted and aliquots of 4 ml each were counted on a Kontron gamma counter for 10 min. The percentage of $^{51}$chromium EDTA excreted over 24 h was then calculated from the formula:

$$\frac{\text{cpm urine}}{\text{cpm standard}} \times \frac{\text{weight of standard}}{\text{weight of dose}} \times \frac{\text{urine volume in mls}}{\text{volume of diluted standard}} \times 100.$$

Creatinine was measured in the same urine sample and a simultaneous blood sample. Creatinine clearance was calculated to estimate renal function.

Thirty-three cancer patients (18 M; 15 F) of mean age 32 yrs (range 10–59 yrs) who had received no cytotoxic treatment for at least 1 month formed the control population. Three of these gave a history of heavy alcohol intake immediately before testing and they were subsequently excluded from the control group (Draper *et al.*, 1983). In three patients, repeated tests were possible before any treatment was given and the results compared.

We applied the test sequentially at 5–8 day intervals in two groups of patients:

1. Nineteen patients (Table I) treated with a single injection of melphalan, i.v. with autologous bone marrow grafting. Fifteen patients were randomly allocated either to receive melphalan 200–200 mg m$^{-2}$ or to receive a priming dose of cyclophosphamide 300–400 mg m$^{-2}$ i.v. followed by melphalan 200–220 mg m$^{-2}$ seven days later. Four patients received 180 mg m$^{-2}$ alone.
2. Two patients treated with cyclophosphamide 7 g m$^{-2}$ i.v. without bone marrow grafting.

### Results

*Acceptability*

The test was generally well tolerated. The EDTA solution is odourless and colourless and none of the untreated patients were unable to take it. After intensive cytotoxic treatment, two patients were completely unable to tolerate the test because of nausea and mouth soreness. Two patients who are already nauseated by their cytotoxic treatment given 5 days earlier, managed to swallow the $^{51}$Cr EDTA but remained nauseated and vomited a few hours after the test began on this occasion and these results are excluded. They completed the test on all other occasions and those results are included in the analysis. In one patient the test failed on day 15 because of nausea. She was very ill with infection, jaundice and fluid imbalance and subsequently died. The completeness of the urine collections in her case was uncertain and she is excluded from analysis.

*Normal range*

In 30 untreated patients, the mean 24 h $^{51}$Cr EDTA excretion in urine was 1.7% of administered dose with standard deviation 0.58% (Figure 1). Three other untreated patients gave a history of heavy alcohol intake and their excretion values were 4.1, 5.6 and 5.9%. These three are excluded from our normal range which extends up to 2.9%. In the three untreated patients in whom the test was done twice the results agreed closely.

*Duration of excretion of $^{51}$Cr EDTA*

In eight patients, urine was collected for 3 days and the daily excretion of $^{51}$Cr EDTA estimated (Figure 2). The excretion fell rapidly and had reached low levels during the third day. However, a significant proportion of the dose was excreted between 24 and 48 h for some patients and so an initial 24 h period of collection may underestimate the total absorption of EDTA in these patients.

*Serial collections after cytotoxic treatment*

The test was performed sequentially after treatment at intervals of ~5 days.

(i) *High dose melphalan (180–220 mg m$^{-2}$) with autologous marrow grafting* The test became abnormal after one week and returned to normal after two weeks (Figure 3). The data suggest that the time of maximum increase in gut permeability was at about day 9. All of these patients experienced diarrhoea after their melphalan treatment, beginning after one and continuing for about one week as described by Cornbleet *et al.* (1983).

**Figure 1** Maximal 24 h urinary excretion of $^{51}$Cr EDTA in patients after i.v. melphalan 180 mg m$^{-2}$ or 200–220 mg m$^{-2}$ compared to untreated controls. The patients treated at the higher dose levels were randomly allocated to cyclophosphamide priming and the primed group have significantly lower $^{51}$Cr EDTA excretion ($P$<0.01 using a $t$ test and Fisher's exact test). Median 24 h excretion in controls was 1.7% compared to median maximal excretion of 3.1% in patients receiving 200–220 mg m$^{-2}$ melphalan with cyclophosphamide priming and 7.4% in patients given the same melphalan dose but no priming. There were no significant differences in timing of studies between the groups.

Gut damage increased with increased melphalan dose and the peak abnormality was significantly greater after melphalan 200 mg m$^{-2}$ than after melphalan 180 mg m$^{-2}$ ($P$=0.05 Wilcoxon Rank Sum Test). Among patients who received 200–220 mg m$^{-2}$ of melphalan, the first 8 received 200 mg m$^{-2}$ and the next 7 received 220 mg m$^{-2}$. They were randomly allocated *within* each dose level to receive a cyclophosphamide prime or not. The maximum measured excretion of EDTA was significantly lower in the patients who received a cyclophosphamide prime (Figure 1, $P$<0.01 $t$ test and Fishers exact test).

**Table I**

| *No. patient* | *Treatment* | *Age* | *Sex* | *Diagnosis* |
|---|---|---|---|---|
| 1–4 | Melphalan 180 mg m$^{-2}$ | 44–60 | F | Breast cancer |
| 5 | Cyclophosphamide priming and melphalan 200 mg m$^{-2}$ | 52 | F | Melanoma |
| 6 | Cyclophosphamide priming and melphalan 200 mg m$^{-2}$ | 42 | F | Breast cancer |
| 7 | Cyclophosphamide priming and melphalan 200 mg m$^{-2}$ | 26 | M | Hodgkin's disease |
| 8 | Cyclophosphamide priming and melphalan 200 mg m$^{-2}$ | 50 | F | Breast cancer |
| 9 | Melphalan 200 mg m$^{-2}$ | 44 | F | Hodgkin's disease |
| 10 | Melphalan 200 mg m$^{-2}$ | 37 | M | Hodgkin's disease |
| 11 | Melphalan 200 mg m$^{-2}$ | 50 | F | Nasopharyngeal cancer |
| 12 | Melphalan 200 mg m$^{-2}$ | 48 | F | Breast cancer |
| 13 | Melphalan 220 mg m$^{-2}$ and cyclophosphamide | 32 | F | Hodgkin's disease |
| 14 | Melphalan 220 mg m$^{-2}$ and cyclophosphamide | 19 | M | Hodgkin's disease |
| 15 | Melphalan 220 mg m$^{-2}$ and cyclophosphamide | 21 | M | Wilms tumour |
| 16 | Melphalan 220 mg m$^{-2}$ and cyclophosphamide | 21 | M | Rhabdomyosarcoma |
| 17 | Melphalan 220 mg m$^{-2}$ | 42 | M | Angiofollicular hyperplasia |
| 18 | Melphalan 220 mg m$^{-2}$ | 42 | M | Hodgkin's disease |
| 19 | Melphalan 220 mg m$^{-2}$ | 21 | F | Soft tissue sarcoma |

**Figure 2** $^{51}$Cr EDTA excretion in three sequential 24 h urine collections after a single test dose in eight patients.

**Figure 3** Twenty-four hour excretion of $^{51}$Cr EDTA after an oral test dose. The test was applied serially following treatment with melphalan and autologous BMT. Four patients given melphalan 180 mg m$^{-2}$ (●); four given melphalan 200 mg m$^{-2}$ (▲); and four given cyclophosphamide 300 mg m$^{-2}$ followed by melphalan 200 mg m$^{-2}$ (○).

(ii) *High dose cyclophosphamide* Two patients received cyclophosphamide 7 g m$^{-2}$ i.v. without bone marrow grafting. No abnormalities were observed in their intestinal permeability.

*Renal function*

Since renal clearance of $^{51}$Cr EDTA might be expected to influence the speed of excretion of the absorbed oral dose, creatinine clearance was measured on each 24 h urine. Change in urine excretion of $^{51}$Cr EDTA in the first 24 h were not explained by changes in creatinine clearance.

### Discussion

The $^{51}$Cr EDTA absorption test was easy to perform and generally well tolerated by our patients even after intensive cytotoxic therapies. $^{51}$Cr edetic acid is cheap, stable and widely used in Nuclear Medicine. The test was reliable and had a narrow normal range although interfering factors such as heavy alcohol intake must be identified (Bjarnason *et al.*, 1984). Collection of urine for longer than 24 h might increase its sensitivity but is time consuming for patients and was not necessary for the purposes of this study.

Abnormalities due to melphalan were detected at doses above 180 mg m$^{-2}$ and seemed to be dose-dependent. The timing of these abnormalities was similar to the timing of histological gut abnormalities after cytotoxic treatment in serial biopsy studies in man (Lubitz & Ekert, 1979; Cornbleet *et al.*, 1983). No intestinal abnormalities were shown after cyclophosphamide treatment which is in keeping with previous evidence for this drug (Shaw *et al.*, 1979).

Although the numbers of patients are not great, we have demonstrated a significant reduction in gut damage after melphalan by cyclophosphamide priming. The effect is quite small and probably not greater than a dose change of 20–40 mg. Its mechanism is not known (Millar & McElwain, 1985) but further investigation of mechanism and of the optimal timing of the priming dose may allow further reduction in gut damage.

We are grateful to Dr Ralph McCready for his support and to the physicians of the Royal Marsden Hospital for allowing us to study their patients.

### References

BJARNASON, I., PETERS, T.J. & VEALL, N. (1983). A persistent defect in intestinal permeability in coeliac disease demonstrated by a $^{51}$CR-labelled EDTA absorption test. *Lancet*, **i**, 323.

BJARNASON, I., O'MORAIN, C. & PETERS, T.J. (1983). Absorption of $^{51}$CR EDTA in inflammatory bowel disease. *Gastroenterology*, **85**, 311.

BJARNASON, I. & PETERS, T.J. (1984). *In vitro* determination of small intestinal permeability: Demonstration of a persistent defect in patients with coeliac disease. *Gut*, **25**, 145.

BJARNASON, I., WARD, K. & PETERS, T.J. (1984). The leaky gut of alcoholism: Possible route of entry for toxic compounds. *Lancet*, **i**, 179.

BJARNASON, I., WILLIAMS, P., ANSELL, B. & PETERS, T.J. (1984). Intestinal permeability in control subjects and patients with rheumatoid arthritis. *Eur. J. Clin. Invest.*, **14**, 45.

CORNBLEET, M.A., McELWAIN, T.J., KUMAR, P.J. & 4 others (1983). Treatment of advanced malignant melanoma with high dose melphalan and autologous bone marrow transplantation. *Br. J. Cancer*, **48**, 329.

CRAFT, A., KAY, H.E.M., LAWSON, D.N. & McELWAIN, T.J. (1977). Methotrexate-induced malabsorption in children with acute lymphoblastic leukaemia. *Br. Med. J.*, **2**, 1511.

DRAPER, L.R., GYURE, L.A., HALL, J.G. & ROBERTSON, D. (1983). The effect of alcohol on the integrity of the intestinal epithelium. *Gut*, **24**, 399.

EDITORIAL. (1985). Intestinal permeability. *Lancet*, **i**, 256.

LUBITZ, L. & EKERT, H. (1979). Reversible changes in duodenal mucosa associated with intensive chemotherapy followed by autologous marrow rescue. *Lancet*, **ii**, 532.

McELWAIN, T.J., HEDLEY, D.W., BURTON, G. & 10 others. (1979). Marrow autotransplantation accelerates haematological recovery in patients with malignant melanoma treated with high dose melphalan. *Br. J. Cancer*, **40**, 72.

MILLAR, J.L., PHELPS, T.A., CARTER, R.L. & McELWAIN, T.J. (1978). Cyclophosphamide pre-treatment reduces the toxic effect of high dose melphalan on intestinal epithelium in sheep. *Eur. J. Cancer*, **11**, 1283.

MILLAR, J.L. & McELWAIN, T.J. (1985). The concept of priming. *Eur. J. Cancer Clin. Oncol.*, **21**, 1303.

SELBY, P., McELWAIN, T.J., CROFTS, M., LOPES, N. & MUNDY, J. (1984). $^{51}$CR EDTA test for intestinal permeability. *Lancet*, **ii**, 38.

SHAW, M.T., SPECTOR, M.H. & LADMAN, A.J. (1979). Effects of cancer, radiotherapy and cytotoxic drugs on intestinal structure and function. *Cancer Treatment Rev.*, **6**, 141.